# “Ninjinto” (Ginseng Decoction), a Traditional Japanese Herbal Medicine, Improves Gastrointestinal Symptoms and Immune Competence in Patients with Chronic Intestinal Failure

**DOI:** 10.1155/2015/462586

**Published:** 2015-10-01

**Authors:** Shuichiro Uehara, Keiko Ogawa, Junsuke Arimitsu, Hiroomi Okuyama

**Affiliations:** ^1^Department of Pediatric Surgery, Osaka University Graduate School of Medicine, 2-2 Yamadaoka, Suita, Osaka 565-0871, Japan; ^2^Department of Kampo Medicine, Kanazawa University Hospital, 13-1 Takara-machi, Kanazawa 920-8641, Japan

## Abstract

*Background*. Treating functional gastrointestinal disorders is extremely difficult. We herein report the effect of the oral administration of Ninjinto (NJT, ginseng decoction), a traditional Japanese Kampo medicine, on chronic intestinal failure. *Patients and Methods*. Seven patients with chronic intestinal failure treated with NJT were evaluated in this study. The primary diseases included chronic intestinal pseudoobstruction (CIPO: *n* = 4), short bowel syndrome (SBS: *n* = 2), and intestinal atresia (*n* = 1). All patients orally received NJT extract granules at a dose of 0.3 g/kg BW per day. The treatment outcomes were then assessed according to the patients' symptoms and consecutive abdominal X-ray findings. *Results*. The targeted symptoms were abdominal distension in four patients, diarrhea in three patients, and frequent hospitalization due to infections in two patients. An improvement in the symptoms was observed in six of the seven patients, whereas one patient with SBS did not show any improvement. An improvement in an abdominal roentgenogram was observed in the four patients with remarkably dilated bowel loops due to CIPO. *Conclusions*. NJT may be effective in controlling functional gastrointestinal disorders associated with chronic intestinal failure. The use of Kampo medicine in the field of pediatric surgery may help to improve the quality of life in children suffering from such conditions.

## 1. Introduction

Pediatric intestinal failure is a complex clinical problem associated with high patient morbidity and mortality. Intestinal failure due to short bowel syndrome (SBS) and/or intestinal dysmotility caused by chronic intestinal pseudoobstruction (CIPO) or a postoperative state of intestinal atresia result in inadequate nutritional absorption to support normal growth and development. Short bowel syndrome (SBS) reflects a state of malabsorption resulting from the loss of a significant portion of the small bowel [1]. In addition, CIPO is a syndrome characterized by episodes mimicking the features of mechanical obstruction in the absence of mechanical, systemic, or metabolic disorders [2]. Managing functional gastrointestinal disorders, especially those associated with intestinal failure, is extremely difficult and hindered by both the poor efficacy and adverse effects of standard pharmacological therapy.

Ninjinto (NJT), a traditional Japanese Kampo medicine, is prepared from four different Chinese crude drugs. This medicine is a major prescription in Japan and most frequently used for the treatment of gastroenteritis, esogastritis, gastric atony, gastrectasis, vomiting, and anorexia [3]. A previous study demonstrated that treatment with Ninjinto not only significantly improves gastrointestinal motility, but also exhibits stronger effects than that observed for various prokinetic drugs, such as cisapride and metoclopramide, in a rat model of postoperative ileus [4]. Therefore, we speculated that NJT may be a candidate as an effective Japanese herbal medicine for ameliorating the symptoms of intestinal failure.

We herein describe our experiences with seven patients with intestinal failure who received therapy with the oral administration of NJT. The aim of this report was to evaluate the effects of NJT in improving intestinal failure and increasing the quality of life (QoL) in these patients by comparing the clinical findings obtained before and after the administration of NJT.

## 2. Patients and Methods

### 2.1. Patients

Seven pediatric patients with intestinal failure due to CIPO, SBS, or intestinal atresia who visited our hospital between April 1, 2012, and April 30, 2014, were selected for this study. All subjects were under 18 years of age or transitioning from pediatric to adult care and continuously used NJT for at least three months. In all cases, no formula changes including modern medical treatment were attempted after the start of the oral administration of NJT.

### 2.2. Evaluation of Efficacy and Safety

We assessed the treatment outcomes and safety based on periodic feedback from the patients and their parents in terms of symptoms, such as abdominal distension, pain, and diarrhea, as well as consecutive abdominal X-ray findings. The primary variables for evaluating the effectiveness of NJT administration included change in the intensity of abdominal complaints from baseline to approximately 3 months following NJT administration and patient satisfaction regarding the improvement of abdominal symptoms, such as abdominal distention and pain and diarrhea. Briefly, the patients were asked to classify the intensity of all abdominal complaints by marking a 100 mm visual analogue scale (VAS) with a straight line before and three months after NJT administration. The VAS assessment was statistically analyzed using a paired *t*-test.

In addition, in order to examine natural killer (NK) cytotoxicity in peripheral blood mononuclear cells (PBMCs) in two patients, measurements of the cytotoxic activity of NK cells were obtained. Briefly, the NK-sensitive cell line K-562, as target cells, was cocultured with PBMCs collected from the peripheral blood of the patients before and three months after the administration of NJT. The percentage of target cells killed by effector NK cells was determined using a ^51^Cr release assay [5].

### 2.3. Dose and Regimen

**Figure 1:**
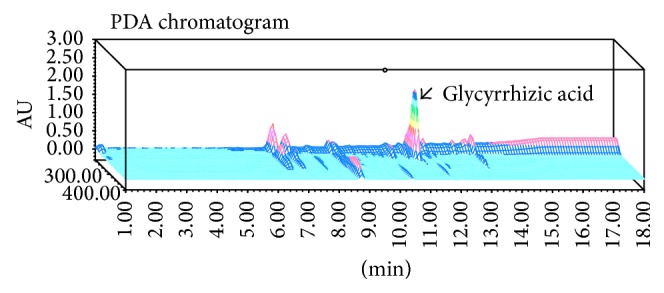
3D HPLC chart of the Kracie NJT extract fine granules manufactured by Kracie (Kracie Holdings, Ltd., Tokyo, Japan). The chemical marker, such as glycyrrhizic acid in the HPLC profile, was identified by comparison with the retention times and UV spectra (210–400 nm) of the reference standards.

All seven patients were administered the NJT extract fine granules manufactured by Kracie (Kracie Holdings, Ltd., Tokyo, Japan) (Table 1) at a dose of 0.3 g/kg BW per day, with a maximum total dose of 7.5 g per day. A 3D HPLC profile of NJT along with a chemical analysis (PDA condition) is shown in Figure 1. As the chemical marker, glycyrrhizic acid was used for quality control.

## 3. Results

A review of the patients is shown in Table 2. The mean age was 13.8 years (1–27), and the diagnoses were as follows: CIPO (*n* = 4), SBS (*n* = 2), and postoperative ileal atresia (*n* = 1). The targeted symptoms of seven patients included abdominal distension and pain in four patients, diarrhea in three patients, and frequent hospitalization due to infections (representing a compromised state) in two patients. No patients withdrew from the oral administration of NJT for at least three months during the treatment period, as the sweet taste of NJT increased patient compliance. No notable adverse effects, such as itching, gastrointestinal symptoms, and other subjective symptoms or abnormalities in the blood count or blood biochemistry data, were observed during or after NJT administration.

**Figure 2:**
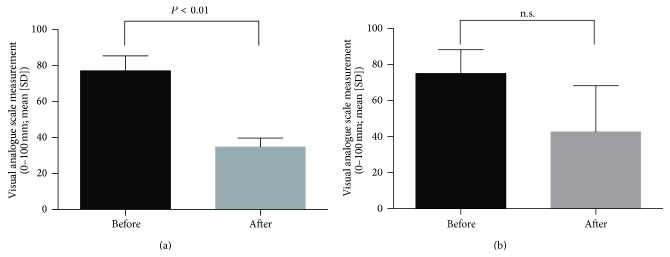
Analyses of the effectiveness of the oral administration of NJT for abdominal symptoms using the visual analogue scale. (a) Abdominal distention and pain (*n* = 4). (b) Diarrhea (*n* = 3) before and 3 months after the oral administration of NJT.

**Figure 3:**
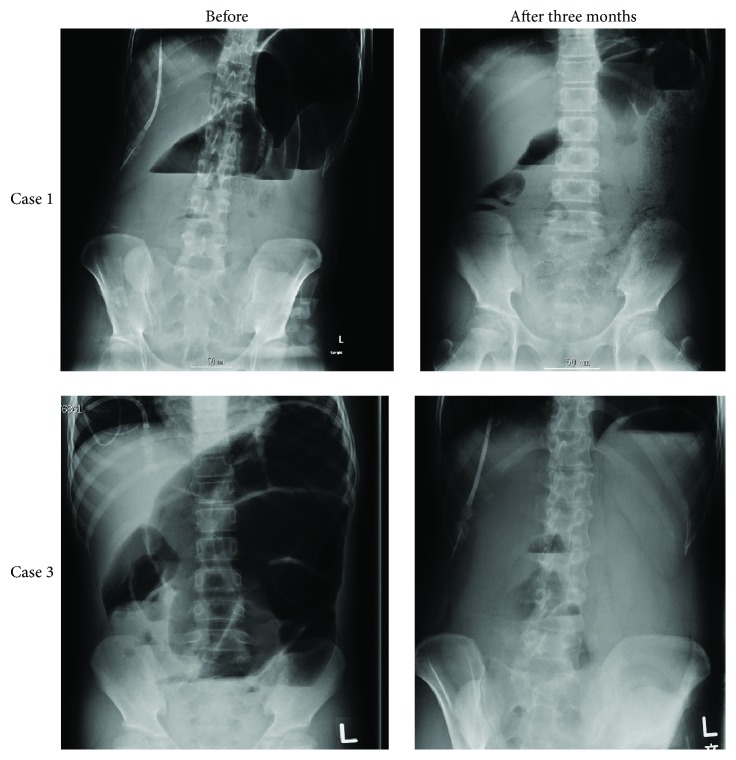
Comparison of abdominal roentgenograms in Cases 1 and 3 obtained before and three months after the oral administration of NJT. Improvements in intestinal dilatation and niveau formation were observed at three months after the oral administration of NJT.

The abdominal distension and pain improved in all four patients, and the intensity of abdominal distension and pain significantly improved on the VAS after 3 months of the oral administration of NJT (Figure 2(a)). Although the VAS after 3 months of the oral administration of NJT did not show a statistically significant decrease of the intensity of diarrhea (Figure 2(b)), diarrhea improved in two of three patients. The frequency of hospitalization due to infections decreased in the two patients (one patient's frequency reduced from six times to twice a year and the other from eight times to twice a year). An improvement in the abdominal roentgenogram findings was observed in the four patients with remarkably dilated bowel loops due to CIPO as shown in Figure 3.

An improvement in the symptoms was observed in six of the seven patients. The patient who did not notice an improvement in his symptoms after the oral administration of NJT did not have remarkable inner cold or qi deficiency, while the other six patients had both of these problems.

The details of two representative cases are described below.

**Case 1 .**

**Figure 4:**
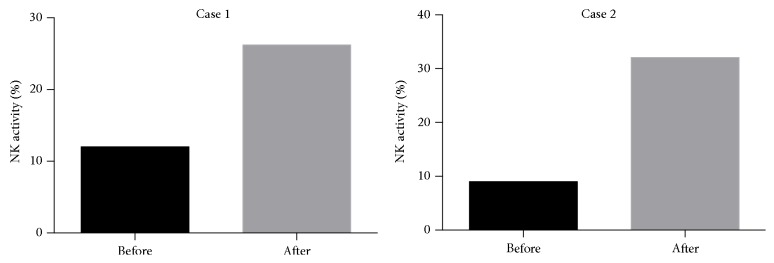
Natural killer activity in the peripheral blood in Cases 1 and 2 before and three months after the oral administration of NJT. All increase in the natural killer cell activity was clearly observed three months after the oral administration of NJT in both Cases 1 and 2. The natural killer cell activity was measured using a ^51^Cr release assay.

A 12-year-old female suffered from repeated abdominal pain, distension, and diarrhea as a result of megacystis-microcolon-intestinal hypoperistalsis syndrome, a condition of CIPO and a representative cause of intestinal failure. Since birth, the patient had been hospitalized on 12 occasions due to periodic abdominal distension three to four times per month and easily developed infections, such as catheter-related bloodstream infections and enteritis. As shown in Figure 3 (upper left), an abdominal roentgenogram showed severe intestinal dilatation and niveau formation, suggesting intestinal obstruction. Neither surgery nor medical treatment was able to successfully control her symptoms. However, after starting treatment with the oral administration of NJT extract, the periodic abdominal distension was well controlled, with evidence of amelioration on an abdominal roentgenogram (Figure 3, upper right) and significant improvements in her general condition and QoL. Interestingly, an increase in the NK activity in the PBMCs was observed three months after the initiation of NJT administration, compared to that seen before the start of NJT treatment (Figure 4, left). The patient has not been hospitalized since receiving oral NJT for two years. Similarly, consecutive improvements in symptoms and abdominal roentgenogram findings following NJT administration were also clearly observed in Case 3 (Figure 3, below).

**Case 2 .**

A 13-year-old male suffered from SBS due to intestinal necrosis associated with gastroschisis at birth. He experienced constant watery diarrhea as well as frequent hospitalization (5-6 times a year) due to bronchitis and pneumonia, probably due to the weakness of his immune system. Based on our experience in Case 1, we speculated that treatment with NJT may increase the NK activity in PBMCs and subsequently decrease the likelihood of infection. One year after starting treatment with the oral intake of NJT extract, the frequency of hospitalization had decreased (to once a year), and the patient's bowel movement disturbance improved. In addition, a blood examination performed three months after the initiation of NJT therapy demonstrated that the NK activity in the PBMCs was upregulated compared to that noted prior to the administration of NJT (Figure 4, right).

## 4. Discussion

Intestinal failure is a complex clinical problem that is associated with high patient morbidity and mortality. Although surgical treatment is often successful, dietary manipulation and pharmacological management of abdominal distension, diarrhea, and a compromised state both have the potential to substantially improve the overall health and quality of life (QoL) of SBS patients [6]. The symptoms of SBS may be caused by multiple etiologies, for example, accelerated intestinal transit, gastric acid hypersecretion, intestinal bacterial overgrowth, and malabsorption of fats and bile salts [6]. Similar to that observed with SBS, most patients with CIPO present with a variety of abdominal symptoms, including abdominal distension, vomiting, chronic constipation, and abdominal pain [7]. It remains still difficult to control these symptoms with medication or surgery in patients with conditions associated with intestinal failure. In six of the seven current patients with intestinal failure, treatment with NJT successfully rescued severe symptoms that could not be managed with existing treatments. Against this background, traditional Japanese herbal medicine (Kampo medicine) may be an attractive alternative option. In fact, Dai-kenchu-to (DKT) was recently reported to be clinically effective in improving gastrointestinal motility in a case of MMIHS [8]. A previous study also demonstrated that NJT not only significantly improves gastrointestinal motility, but also shows stronger effects than those of various prokinetic drugs, such as cisapride and metoclopramide, in a rat model of postoperative ileus [4], and NJT has been confirmed to increase the level of motilin, a powerful inducer of gastrointestinal motor activity, and somatostatin, which participates in the regulation of gut motility by exerting both inhibitory and stimulatory actions in human plasma. Sato et al. reported that NJT significantly increases the release of plasma CGRP, substance P, motilin, and somatostatin, thereby contributing to the gastrointestinal protection, motility, and secretion [3]. For these reasons, NJT is thought to be effective in treating gastrointestinal symptoms and obtaining an improvement in the abdominal roentgenogram findings in patients with intestinal failure.

Surprisingly, in the present study, the administration of NJT increased the NK activity in PBMCs in two patients, who consequently became relatively insusceptible to treatment. There are several hypotheses explaining this phenomenon. First, in an animal study, Kaga et al. confirmed that NJT augments the NK activity in vivo, and the authors suggested that certain components of NJT, which contains four different components, directly stimulate immune system in vivo [9]. Another explanation is that the increase in QoL resulting from the improvements in abdominal symptoms indirectly contributes to increasing the NK activity.

Panax ginseng is one of the most important crude drugs in NJT. Ginseng contains ginsenoside Rb1 as its main constituent and is traditionally used in Kampo formulas for cancer, inflammation, stress, and ageing. Ginsenoside Rb1 present in orally administered ginseng is metabolized to bioactive compounds, including compound K, by gut microbiota prior to absorptions in the blood. Colonic bacteria cleave the oligosaccharide connected to the aglycone compound stepwise from the terminal sugar to yield the major metabolites [10]. The intestinal bacterial metabolism of ginseng, particularly ginsenoside Rb1, may be dependent on the composition of gut microbiota, such as* Ruminococcus* spp.,* Bacteroides* spp., and* Bifidobacterium* spp. [11], and the continued administration of NJT may induce the development of a suitable composition of gut microbiota for the metabolism of ginsenoside. The other important crude drug in NJT is* Atractylodes japonica*. It has been reported that SKI3246 of the rhizome of* Atractylodes japonica* is potentially effective in treating visceral hypersensitivity in experimental rat model of irritable bowel syndrome [12]. According to the Kampo diagnosis, NJT improves interior cold, a pathological state characterized by the preponderance of yin cold or a decline in yang qi in the interior, thus tonifying the digestive system. The intestinal failure can be considered to be a deficiency of spleen qi, a pattern marked by digestive dysfunction such as the loss of appetite, abdominal distention, or loose bowels. For this reason, NJT is one of the best formulas for patients with intestinal failure associated with interior cold.

In this study, NJT was administrated to pediatric patients with intestinal failure without the Kampo diagnosis because most of the pediatric patients with intestinal failure could be considered to have the disease pattern of NJT. As a result, one of the patients with SBS without the disease pattern of NJT, such as inner cold or qi deficiency, did not have a symptomatic improvement following NJT treatment. It is important to determine the cause, nature, and location of the pathological change at specific stages of the disease by the Kampo diagnosis for case selection in order to obtain sufficient effects. In future investigation, the selection criteria according to the Kampo diagnosis should be included in the selection of the patients. It may be possible to find inner cold in the diagnostic process with an interview, for example, and whether the patient's symptoms exacerbate with cold stress.

Similar to that observed in our cases, various Kampo formulas have been prescribed for pre- and postoperative pediatric patients with gastrointestinal anomalies and diseases. Representative Kampo formulas used for daily treatment in the pediatric surgical field include rikkunshito after upper gastrointestinal surgery [13], daikenchuto after colorectal surgery [14], inchinkoto for postoperative biliary atresia, and hainosankyuto for perirectal abscesses [15]. These formulas have been reported to achieve satisfactory results. Therefore, we believe that Kampo formulas have the potential to greatly contribute to controlling perioperative symptoms and preventing permanent damage in the field of pediatric surgery.

Kampo medicine is in general very safe. There are few adverse effects reported in connection with some Kampo formulas, such as pneumonitis, pseudoaldosteronism, and liver dysfunction. Nevertheless the patient's condition should be continuously monitored during treatment using careful history-taking, laboratory assessments, and physical examination. Since in Japan Kampo medicines are prescribed by medical doctors and are monitored by the national pharmacovigilance system, safe use can be insured.

## 5. Conclusion

NJT may be effective in controlling the symptoms of intestinal failure, CIPO, and SBS, and further randomized controlled trials are warranted to evaluate the clinical efficacy of NJT for patients with these disorders. The use of Kampo medicine in the field of pediatric surgery may contribute to improving the quality of life in children suffering from such conditions.

## Figures and Tables

**Table 1:** Composition of the NJT extract granules manufactured by Kracie (Kracie Holdings, Ltd., Tokyo, Japan).

Ingredient	Volume (g)^*^
JP processed ginger	3.0
JP *Glycyrrhiza *	3.0
JP *Atractylodes japonica* rhizome	3.0
JP ginseng	3.0

^∗^6.0 g of Kracie NJT extract fine granules contains 3.0 g of a dried extract of the above mixed crude drugs. JP: Japanese Pharmacopoeia.

**Table 2:** Review of pediatric patients with intestinal failure treated with NJT.

Case number	Gender	Age	Diagnosis	Targeted symptoms
1	F	12	CIPO	Abdominal distention & pain
2	M	13	SBS	Diarrhea & compromised state
3	F	27	CIPO	Abdominal distention & pain
4	M	10	CIPO	Abdominal distention & pain
5	M	28	CIPO	Abdominal distention & pain
6	M	1	Ileal atresia post-OP	Diarrhea & compromised state
7	M	6	SBS	Diarrhea

CIPO, chronic intestinal pseudoobstruction; SBS, short bowel syndrome; OP, operation.
